# Race Against Antimicrobial Resistance Requires Coordinated Action – An Overview

**DOI:** 10.3389/fmicb.2015.01536

**Published:** 2016-02-02

**Authors:** J. Premanandh, B. S. Samara, A. N. Mazen

**Affiliations:** Central Testing Laboratories, Quality and Conformity Council Abu Dhabi, UAE

**Keywords:** antimicrobials, resistance, stewardship, growth promotors, therapeutic use

## Abstract

Resistance developed by microbes is challenging success stories of treatment of infectious diseases with anti-microbials. Developing new antimicrobials against these resistant organisms does not progress at the same speed. In an effort to address this key issue, this work overviews the role of different stakeholders and discusses preventative and control measures for effective management of available resources. Roles and concerns of physicians, pharmacists and the public are also discussed. More than anything, this situation requires immediate action to establish antimicrobial stewardship program, control over the counter sale and promote public awareness. The paper also confronts the idea of curbing the use of antimicrobials using mass media, while detailing the consequences of non-therapeutic use. The role of policy makers in taking global action is essential to establishing authority or agency for formulating national guidelines and regulations for prudently using antimicrobials. To do this, this paper recommend the establishment of a global fund. In conclusion, the race against resistance is a collective responsibility requiring coordinated action at local, national, regional and international levels to ensure sustained utilization of antimicrobials.

## Introduction

Antimicrobials are naturally occurring or synthetic chemical agents that kill or inhibit the growth of microorganisms. They are one of the most successful forms of chemotherapy used in the treatment of infectious diseases. Although evidence suggests the existence of antimicrobials back to 350–550 CE (Bassett et al., 1980; Nelson et al., 2010), their effective use began in the mid-1940s making revolutionary changes in treating infectious diseases. They remain the method of choice for such treatment. However, their success stories are being challenged by resistance development due to misuse/overuse of antimicrobials. Though observed even before the first clinical use of penicillin in the early 1940s (Wright, 2010); the current situation on resistance to almost all the antimicrobial agents poses alarming risks to human and animal health worldwide.

Microbial resistance is a natural evolutionary processual response to selective pressure (Wright, 2010). As a result, antimicrobial agents fail to act against microbes (bacteria, fungus, parasites, or viruses) resulting in persistent infection. Microbes acquire resistance by the incorporation of resistance genes into the microbial genome or plasmids through conjugation, transformation, or transduction (Tenover, 2006). It also occurs due to spontaneous mutations at the locus that control drug susceptibility. Although it is a natural process, excessive and prolonged use facilitates resistance and creates a serious threat.

Antimicrobial consumption increased drastically between 2000 and 2010 (World Health Organization [WHO], 2014a). In particular, BRICS countries (Brazil, Russia, India, China, and South Africa) recorded the highest usage at around 76% with an overall global increase of 36%. Between 2010 and 2030, antimicrobial consumption is expected to increase by 67% worldwide. In particular, the amount of antimicrobials given to farm animals will likely increase by two-thirds over the next 15 years without intervention (Van Boeckel et al., 2015). Studies have found that countries with high consumption rates also have high resistance rates (Ferech et al., 2006). To this end, developers of new antimicrobials against these resistant microbes have failed to progress at the same speed. In fact, only three new classes of antimicrobials have been introduced since 2000 for human use (Spellberg et al., 2008). Resistance is viewed as a global threat and therefore, necessitating control to preserve the efficacy of antimicrobials by all possible means to continue to reap the benefits traditionally associated with them.

Given this background, this work provides an overview of the role of different stakeholders and discusses preventive and control measures for effective management to minimize the development of resistance.

## Controlling Therapeutic Overuse

In general, antimicrobials are received for therapy or prophylaxis during the course of infection, but at least one-half of patients are receiving them unnecessarily or inappropriately (Hogerzeil, 1995). For instance, the most common cause of infections of the upper respiratory tract is associated with viral agents (Zoorob et al., 2012), where antimicrobials are ineffectual. Nevertheless, general practitioners often prescribe antimicrobials for this ailment (Bagger et al., 2015).

Studies from the Gulf Cooperation Council countries (Bahrain, Kuwait, Oman, Qatar, Saudi Arabia, and the United Arab Emirates) indicated that inappropriately prescribing and overusing, including self-medication, appears to be the main cause of resistance development (Memish et al., 2007; Aly and Balkhy, 2012). However, from the physician’s perspective, it is difficult to diagnose in the early stages whether an infection is viral or bacterial, especially in the cases of upper respiratory tract infections and diarrhea (Kotwani et al., 2010). Behavioral characteristics also play a crucial role in misuse and/or overuse. For instance, patients sometimes demand medication on the basis that it was effective in previous illnesses – physicians may comply.

The role of pharmacists in dispensing over the counter (OTC) sale of antimicrobials is noteworthy. By law, it is prohibited in many countries (Wirtz et al., 2013) but is nevertheless poorly enforced and results in unlicensed distribution (Pan American Health Organization [PAHO], 2004). While each stakeholder plays a key role in restricting usage, according to a new survey from the World Health Organization (WHO), pharmacists are among the best positioned to promote appropriate use of antimicrobials; they can play a crucial role in combating resistance along with policy-makers and health practitioners (World Health Organization [WHO], 2014b). Encouraging pharmacists through awareness programs and incentives would arrest the point of sale. Achieving compliance with OTC laws, stricter enforcement and monitoring measures should all be applied. There must also be severe penalties for non-compliant pharmacies. An example of this is the retention of the operating manager’s license or closure of the business in the most extreme of circumstances.

The Infectious Disease Society of America and the Society for Health Care Epidemiology of America published guidelines for antimicrobial stewardship in 2007 to establish better management of clinical treatment resources. These guidelines aim to provide information on how to establish such programs within health care institutions (Dellit et al., 2007). The primary goal is to prevent or slow down resistance – these guidelines are a logical first step to facing the issue. Results from previous studies suggest that the systematic stewardship program not only helps to curb unnecessary usage, but also reduces the treatment cost considerably. In one study, the program saved approximately US$ 1 million in 18 months (Bantar et al., 2003). Similarly, Goff et al. (2012) showed that they achieved annual cost savings of $832,590. Many countries have established national stewardship programs and experienced beneficial impacts even in resource-limited settings.

**FIGURE 1:**
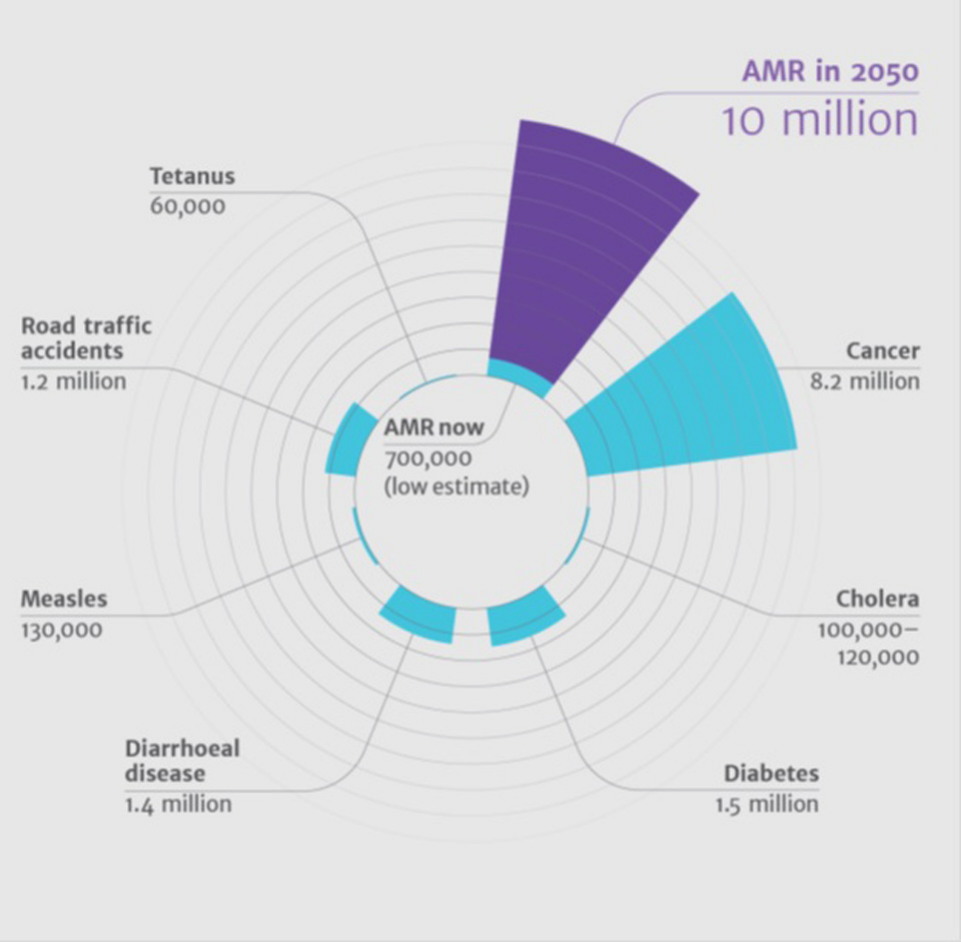
**Antimicrobial death estimate in 2050 compared to mortalities caused by other major illnesses.** Source: Review on Antimicrobial Resistance, 2014. Antimicrobial resistance: tackling a crisis for the health and wealth of nations. Review on Antimicrobial Resistance, London, UK.

Such a program is not the only way to combat mis- and overuse of antimicrobials. Mass media campaigns in Belgium resulted in a 36% decline in antimicrobial prescriptions and prompted France to initiate a similar campaign directed toward the public and general practitioners, particularly in the implementation of rapid strep diagnostic tests for upper respiratory infections (Sabuncu et al., 2009). In general, education and awareness lead to better knowledge and also predicts positive attitude (Awad and Aboud, 2015). Countries lacking such initiatives need to implement media campaigns to educate the public, while stewardship programs can help physicians in hospitals to curb the unwanted use of antimicrobials. Countries like India have many private practitioners as an important source of medical care, especially in rural areas. Awareness programs targeting these practitioners should therefore be considered since they are generally the first contact preference for medical care in a local community (Rao, 2005). Thus, effective programs targeting the reduction of antimicrobials at all levels should be considered failing which would result in a higher number of deaths (World Health Organization [WHO], 2014a) in comparison to mortalities caused by other major illnesses (**Figure 1**).

## Controlling Non-Therapeutic Use

Initially, antimicrobials were administered to animals for prophylactic and curative reasons. In the mid-1950s, small quantities of antimicrobials such as penicillin and tetracycline were found to enhance the feed-to-weight ratio and increase growth rate in poultry, swine, and beef while reducing mortality and morbidity (Stokestad and Jukes, 1950). As a result, the Food and Drug Administration of the United States (US FDA) approved these drugs in 1951 (Jones and Ricke, 2003). This resulted in the extensive use of anti-microbial drugs in farm animal practice. The adverse effect of the non-therapeutic use of antimicrobials was not realized until Smith and Crabb (1957) reported that diets containing tetracycline in pig and chicken resulted in tetracycline resistant *Escherichia coli*. Studies quickly proceeded to elucidate the impact of the non-therapeutic use of antimicrobials in farm animals (Langlois et al., 1984; Aarestrup, 1999; Funk et al., 2006).

Although misuse and over-use in both human medicine and intensive animal production have been recognized as one of the important factors in the current crisis of resistance, non-therapeutic use has led to multi-drug resistance on agents never previously associated with farms (Levy, 2002). As a result, the first ban on farm use of antimicrobials as growth promoters was enacted in 1986, in Sweden followed by Denmark, United Kingdom, and other countries of the European Union (Cogliani et al., 2011). However, many countries refuse to take a firm position, despite the European experience that the ban has reduced overall consumption in animals (Casewell et al., 2003).

On the contrary, proponents argue that the withdrawal of small quantities of antimicrobials resulted in heavy use of antimicrobials to tackle the emergence of pathogens like *Salmonella* sp. and *Campylobacter* sp. (Hao et al., 2014). This has made some countries skeptical about the ban. Infectious diseases’ pervasiveness is generally linked to poor hygiene and management practices. For instance, strong sanitation and hygiene procedures along with biosecurity measures helps alleviate antimicrobials in addition to health checks and vaccination programs. Likewise, frequent visits and checks by animal health professionals ensures the well-being of animals and is a key element in successful animal husbandry. However, in reality antimicrobials are often used in farm animals with little or no veterinary consultation. A survey in the USA reported that only 42% of pig farms used the services of veterinary medical practitioners. It is noteworthy to point out the role of veterinary medical practitioner here. For instance, not all sick animals are infected, nor do all infections require treatment with antimicrobials (Weese et al., 2015). In this way, correct prognoses and diagnoses are primary elements in decision making regarding their usage. This rests ideally with veterinarians on the basis of symptoms and appropriate laboratory investigation, including culture and sensitivity tests as they pertain to individual animals or groups. Such practices not only protect the animals against diseases, but also provides economic benefits. The animal health practitioner is responsible for educating farmers and ensuring that routine prophylactic use of antimicrobials should never be a substitute for good animal health management. For instance, good weaning practices and avoiding excessive herd or flock size reduces stress and improves animal’s natural ability to fight infectious diseases without assistance (Humphrey, 2006).

## Role of Policy Regulation in Preserving Antimicrobials

Policy-makers play a key role in tackling the fast-emerging public health problem of resistance. The WHO has declared antimicrobial resistance as a global concern, insisting national surveillance systems. Harmonized integrated surveillance of antimicrobial resistance has been implemented in a limited number of countries, despite several international recommendations during the last two decades (World Health Organization [WHO], 2014a). Poor laboratory capacity, infrastructure, and data management prevent effective surveillance, making it difficult to discern patterns of resistance and identify disease trends and outbreaks in many countries. Each country should therefore establish an authority or agency to formulate national guidelines and regulations in an effort to preserve the efficacy of antimicrobials. Measures should be consistent in these programs with the existing international guidelines and codes of practice such as the Codex Guidelines for Risk Analysis of Foodborne Antimicrobial Resistance (CAC/GL 77-2011) (FAO/WHO, 2011), and the Codex Code of Practice to minimize and Contain Antimicrobial Resistance (CAC/RCP 61-2005) (FAO/WHO, 2005). Decision makers must terminate non-therapeutic use of antimicrobials that are also used in treatment of humans due to mounting evidence that it contributes to antimicrobial resistance development and endangers humanity.

Continuous surveillance and data integration of microbial isolates are key components in assessing the current status of resistance. The World Organization for Animal Health (OIE) has developed standards toward this purpose that help to detect the emergence and spread of resistant microbes that may cause foodborne disease (World Health Organization [WHO], 2014b). Establishing surveillance and monitoring programs therefore addresses two key issues: early warning and drug resistance. Developed nations may lend financial/technical support and help to develop a set of action plans to combat rapidly developing antimicrobial resistance in coordination with the WHO.

International financial institutions need to organize to develop a global fund toward creating an enabling environment for underdeveloped and developing countries to implement effective, evidence-based programs. Since many countries lack basic surveillance and laboratory capacity, technical, and monetary support is crucial to creating a culture of awareness and battling antimicrobial resistance.

## Current Status, Achievements, and Challenges

In an effort to address this alarming issue, the US FDA has established a list of “qualifying pathogens” with the potential to pose a serious threat to public health. Generating Antimicrobial Incentives Now (GAIN) has been signed as part of the Food and Drug Administration Safety and Innovation Act to encourage development of new antibacterial and antifungal drugs. These are required to treat serious or life-threatening infections, whereas the Act provides incentives like designating eligibility as fast-track products, or an additional 5 years of exclusivity to be added to certain exclusivity periods (Food and Drug Administration, 2014).

As of March 2015, 36 new antimicrobials with a potential to treat serious bacterial infections are under various stages of clinical trials. Nevertheless, only 60% of drugs entering phase 3 get approvals, resulting in about 5 new drugs or so. Recently, the US FDA approved three new antimicrobials: dalbavancin, oritavancin, and tedizolid to treat patients with bacterial infections (The PEW charitable Trusts, 2015). While new drugs are being developed to meet the current crisis, resources need to be allocated to meet the twin challenges of preserving the existing antimicrobials and developing new ones. Social media networking can contribute to preserving existing antimicrobials. By targeting active users, it is possible to communicate consequences of inaction more frequently, efficiently, and on an unignorably mass scale. As an example of a social media campaign, a recent report from South Africa showed Twitter as a useful tool to improve awareness of antimicrobial resistance (Goff and van den Bergh, 2015).

The World Health Assembly recently endorsed a global action plan with five major objectives, including: better awareness and understanding of antimicrobial resistance, strengthening surveillance and research, reducing the incidence of infection, optimizing the use of antimicrobial medicines and ensuring sustainable investment in countering antimicrobial resistance (World Health Organization [WHO], 2015). It is the responsibility of member states to initiate the plan and monitor its success.

Several initiatives are also being made specifically to combat non-therapeutic use, including the US FDA’s proposal to phase out use over the course of three years. Companies have indicated their compliance, despite it being only an unenforced recommendation (Food and Drug Administration [FDA], 2015). The companies have agreed to change the marketing status of their products from OTC to Veterinary Feed Directive or prescription.

A recent report from an OIE survey shows that 51% of 152 participating countries have completely banned growth promoters, while 19% have committed to a partial ban (Rushton, 2015). Although legislation, surveillance systems, and bans on non-therapeutic use are on the rise (Diaz, 2013), complete elimination of this type of use is impossible without alternative solutions.

Using antimicrobials for growth promotion generates more profit than their use for disease control. Hence, it has been a common practice in livestock sector for the last half century. However, in the case of good housing and hygiene, differences in the growth rates were insignificant between consumers and non-consumers of antimicrobials (Rushton, 2015). In some cases as in the case of poultry, American farmers have actually experienced economic loss when using antimicrobials (Graham et al., 2007). It is therefore obvious that antimicrobials have little impact as growth promoters and can in general be substituted with better and more hygienic living conditions. In the case of farm animals, whole herd health is the ultimate goal and to prevent infectious disease, it is common to medicate the drinking water of the whole herd, a commonly recognized means animals receive infectious agents. Alternative treatments have proven effective in treating farm water quality. Ozone is an extremely reactive oxidant and a very effective sanitary agent in the food and agricultural industries (de Candia et al., 2015; Pierron et al., 2015). This would be an effective substitute to antimicrobials. Although it is difficult to substitute antimicrobials in animal husbandry and, prudent use is the only choice, possible measures to minimize their use at all levels should be applied.

**FIGURE 2:**
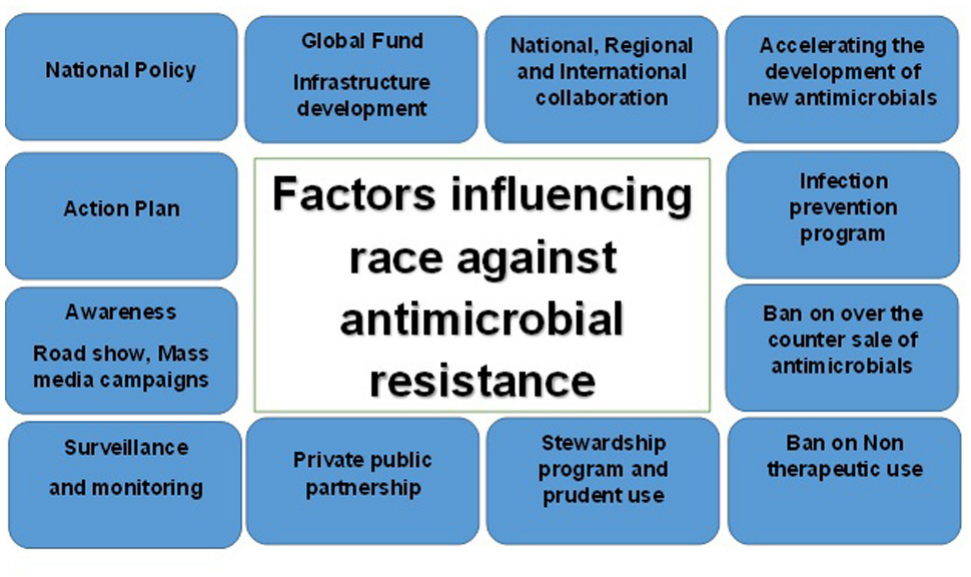
**Factors influencing race against antimicrobial resistance**.

Research and development of new antimicrobials are yet another vital area progressing at a slower rate because of challenges encountered by pharmaceutical companies. Regulatory obstacles and economic disincentives force companies to focus on more lucrative areas like cardiology and oncology (Bettiol and Harbarth, 2015). International groups have been aiming to create and test a new economic model for antimicrobial research autarky. Sooner or later, such models should accelerate research and development of new classes of antimicrobials to control infectious agents. In an era of mounting antimicrobial resistance, priority among researchers needs to be targeted toward novel agents that circumvent, or at least, minimize resistance. **Figure 2** depicts the race against resistance and how it is a complex issue facing a plenitude of factors; as a result, every stakeholder must intervene by all possible means in order to manage this crisis.

## Concluding Remarks

Antimicrobial resistance is an alarming threat to global health (Laxminarayan et al., 2013), where 10 million deaths are estimated to occur annually by 2050 without appropriate intervention. The WHO has drafted a sector-wide action plan to be implemented globally to attempt this. Considering the current situation, it is inevitable to establish legal frameworks and monitoring programs on regional and national levels to ensure appropriate usage of antimicrobials, which also means reducing their use drastically. Governments and NGOs alike must develop nationwide education and awareness programs, while regulatory authorities should monitor the use of antimicrobials in hospitals and health care centers. Incentive programs may be incorporated to encourage appropriate usage, which will trigger stakeholders’ compliance.

Antimicrobial use as growth promoters need to be terminated. Quality management programs in farms should be enforced to prevent infection by improving sanitation, cleaning up water supplies, etc. In addition, accelerating antimicrobial development needs to be a priority. International collaboration and capacities for prevention, surveillance, and control of antimicrobials should be enhanced. In conclusion, racing against antimicrobial resistance is a collective responsibility requiring coordinated action at all levels to ensure sustained benefits of antimicrobials and head off any threats to humanity’s future.

## Author Contributions

PJ conceived the idea and wrote the manuscript. SS and MN provided additional insights on policy regulation and current status and reviewed the manuscript and involved in the revision with additional information.

## Conflict of Interest Statement

The authors declare that the research was conducted in the absence of any commercial or financial relationships that could be construed as a potential conflict of interest.
